# Assessing Social Capital among Chinese Older adults: Dimensions and Associative Factors

**DOI:** 10.1093/geroni/igaf122.2451

**Published:** 2025-12-31

**Authors:** Yuekang Li

**Affiliations:** University of Chinese Academy of Social Sciences, Beijing, Beijing, China (People’s Republic)

## Abstract

Recent research highlights social capital as a crucial factor in healthy aging, with its impact varying across contexts and cultures. This study delves into the nature of social capital among older adults in China, examining its dimensions and the influence of individual and environmental characteristics. Utilizing the 2016 wave of the China Family Panel Study, the research employed factor analysis and Multiple indicators, multiple cause (MIMIC) structural equation models to dissect the components of social capital within this group. The analysis identified three key levels of social environment—family, community, and macro society—that shape the social capital of Chinese older adults. The study’s findings emphasize the complex interplay between individuals and their social environments. It was observed that those with disabilities tend to have enhanced family-level social capital but diminished community-level social capital. This suggests that while families remain a crucial support system, reliance on them may limit access to broader social resources. Moreover, the community’s physical environment emerged as a significant factor in determining social capital, underscoring the importance of neighborhood conditions in fostering social connections among older adults. These findings reveal the multifaceted nature of social capital in later life and highlight the need for targeted policy and practice interventions. By focusing on individuals with high family-level social capital, efforts can be made to reduce vulnerabilities and improve the capacity of older adults to engage with wider social networks, thereby enhancing their overall well-being and ability to adapt to diverse environments.

